# Association of the Expression Level of miR-16 with Prognosis of Solid Cancer Patients: A Meta-Analysis and Bioinformatic Analysis

**DOI:** 10.1155/2020/8815270

**Published:** 2020-07-25

**Authors:** Wanting Zhang, Feixiang Zhou, Danjie Jiang, Yingying Mao, Ding Ye

**Affiliations:** ^1^Department of Epidemiology and Biostatistics, School of Public Health, Zhejiang Chinese Medical University, Hangzhou, 310053 Zhejiang, China; ^2^Ningbo Municipal Center for Disease Control and Prevention, Ningbo, 315010 Zhejiang, China

## Abstract

**Objective:**

To assess the association between the expression level of miR-16 and prognosis of solid cancer patients by meta-analysis and bioinformatic analysis.

**Methods:**

PubMed, Web of Science, and Embase databases were searched until October 31, 2019, to identify eligible studies reporting the association of the miR-16 status with the prognosis of solid cancer patients. Hazard ratios (HRs) with 95% confidence intervals (CIs) were pooled, and a heterogeneity test was conducted. Sensitivity analysis and a publication bias test were also carried out. Furthermore, the miRpower database was used to validate the association.

**Results:**

Thirteen articles with 2303 solid cancer patients were included in the meta-analysis. Solid cancer patients with low expression level of miR-16 had shorter survival time (*I*^2^ = 84.0%, HR = 1.47, 95% CI: 1.13-1.91, *P* = 0.004). In the subgroup analyses of cancer sites, low miR-16 expression level was associated with poor prognosis in the reproductive system cancers (*I*^2^ = 33.3%, HR = 1.24, 95% CI: 1.06-1.45, *P* = 0.008). Sensitivity analysis suggested that the pooled HR was stable and omitting a single study did not change the significance of the pooled HR. Begg's test and Egger's test revealed no publication bias in the meta-analysis. In bioinformatic analysis, the significant association between miR-16 level and prognosis of patients with reproductive system cancers was further confirmed (HR = 1.21, 95% CI: 1.03-1.42, *P* = 0.017).

**Conclusion:**

Low expression level of miR-16 is an indicator for poor prognosis of solid cancer patients, particularly in reproductive system cancers.

## 1. Introduction

MicroRNAs (miRNAs), a family of 21-25-nucleotide small noncoding RNAs, participate in a variety of pathophysiological processes, such as cell migration, invasion, proliferation, and differentiation [1]. They regulate gene expression and function as oncogenes or tumor suppressors posttranscriptionally by degrading target miRNAs or blocking their translation [2]. Therefore, the abnormal expression of miRNAs was found in patients with a variety of solid cancers, such as breast cancer [3, 4], prostate cancer [5, 6], and colorectal cancer [7]. Besides, dysregulated expression of miRNAs could result in solid cancer progression and might serve as an independent predictor for solid patient outcomes [8]. For example, miR-125b was an independent prognostic marker for lung cancer [9], and miR-221 was a predictor of prognosis of patients with hepatocellular carcinoma [10].

miR-16 has been cloned by independent groups, and this precursor sequence maps to chromosome 13 [11]. Numerous studies have shown that miR-16 played a role in carcinogenesis and affected the occurrence of solid cancers. The evidence from a large-scale population-based study showed that circulating miR-16 could act as a biomarker in cancer detection, though miR-16 expression level was different in various solid cancers [12]. In addition, a systematic review and meta-analysis came to the conclusion that miR-16 family members had a high application value in the diagnosis of solid cancers [13].

As for the association between miR-16 and prognosis of solid tumors, the results of previous studies remain controversial. A considerable proportion of studies reported that solid cancer patients with low expression level of miR-16 had a shorter survival time, including gastric cancer, ovarian cancer, colorectal cancer, and oral squamous cell carcinoma [14–17]. However, several studies reported that high miR-16 expression predicted poor overall survival in patients with colorectal cancer and esophageal cancer [18, 19]. Therefore, we conducted a meta-analysis to systematically evaluate the prognostic value of the expression level of miR-16 for solid cancer patients. Moreover, we used the miRpower database to validate and complement the meta-analysis.

## 2. Materials and Methods

The study was registered in the International Prospective Register of Systematic Reviews (PROSPERO: CRD42020139877). The PRISMA checklist for reporting the meta-analysis is shown in Supplementary Table 1.

### 2.1. Search Strategy and Selection Criteria

The keywords and subject terms used were “miR-16 OR microRNA-16 OR hsa-miR-16 OR miRNA-16” AND “cancer OR tumor OR carcinoma OR neoplasm OR melanoma” AND “survival OR survive OR subsistence OR prognosis OR prognostic OR progression OR development OR outcome OR recurrence OR mortality”. PubMed, Web of Science, and Embase databases were searched for relevant studies published within the period from the establishment of the database to October 31, 2019.

The studies which met the following explicit criteria were included: (1) the study design was a prospective study, (2) the study population were patients who have been diagnosed with certain cancers by medical institutions, (3) miR-16 expression levels were classified as two categories, (4) hazard ratio (HR) and 95% confidence intervals (CIs) can be extracted directly or indirectly by calculation, (5) types of cancer are limited to solid cancer, and (6) language is limited to English.

The exclusion criteria were as follows: (1) systematic reviews or meeting abstracts or letters, (2) the research objects being only plant or animal models, (3) duplicate studies retrieved from various databases, (4) miR-16 expression levels being classified as three or four categories, (5) the outcome not recording patient survival, and (6) HR and 95% CI which were not provided or could not be calculated.

### 2.2. Data Extraction and Quality Assessment

Two authors (WZ and FZ) extracted the following information from the included studies: first author, year of publication, sample size, age and gender distribution of the study population, sample types, cancer sites and stages, follow-up period, statistical methods, and HR with 95% CI and *P* value. Survival time is defined as the total length of time from diagnosis with cancer or cancer treatment intervention to the death date or the end of the follow-up.

Two researchers (DJ and YM) referred to the tumor marker guidelines for prognostic studies [20] to conduct quality evaluation and then checked results. The guideline consisted of 20 items in four parts: introduction, materials and methods, results, and discussion, totaling 20 points. The higher score indicated higher quality of the study.

### 2.3. Bioinformatic Analysis

The publicly available database miRpower (http://www.kmplot.com/mirpower) was used to further validate and supplement the meta-analysis [21], which is able to analyze miRNA-derived survival outcome signatures dynamically for one or more types of solid cancer. We also pooled the associations between mir-16 expression and survival of the same system of solid cancers to obtain an overall estimate. *P* < 0.05 was considered statistically significant.

### 2.4. Statistical Analysis

The STATA version 14.0 (Stata Corp.) was used in statistical analyses. The association between miR-16 and prognosis of patients with cancers was evaluated by HR with 95% CI. If HRs were not directly reported in the included studies, they were estimated based on the number of two comparable groups and the *P* value calculated by log-rank by the method which was described by Tierney et al. [22], and the 95% CI of the HR was estimated according to the method described by Altman and Bland [23]. In addition, high expression of miR-16 was used as the control group. The cutoff value was defined by study-specific reference ranges.

A heterogeneity test was carried out by the Cochran *Q* test and *I*^2^ statistic. A fixed effects model was applied if the *P* value of the *Q* test was ≥0.10 and the *I*^2^ statistic was <50%; otherwise, the random effects model was used. The sources of heterogeneity were analyzed through subgroup analyses and metaregression analyses. Subgroup analyses were carried out stratified by publication year, cancer site, region, quality score, sample size, statistical method, and biosample. By using the regression method for meta-analysis, these variables can be added to the analysis to reduce the variance that cannot be explained [24].

We performed sensitivity analysis to assess whether a particular study may influence the summary risk estimate, in order to investigate the robustness of our main analysis. Publication bias was assessed by Begg's test [25] and Egger's test [26], and funnel plots were constructed to intuitively reflect the bias.

## 3. Results

### 3.1. Study Selection and Characteristics

**Figure 1:**
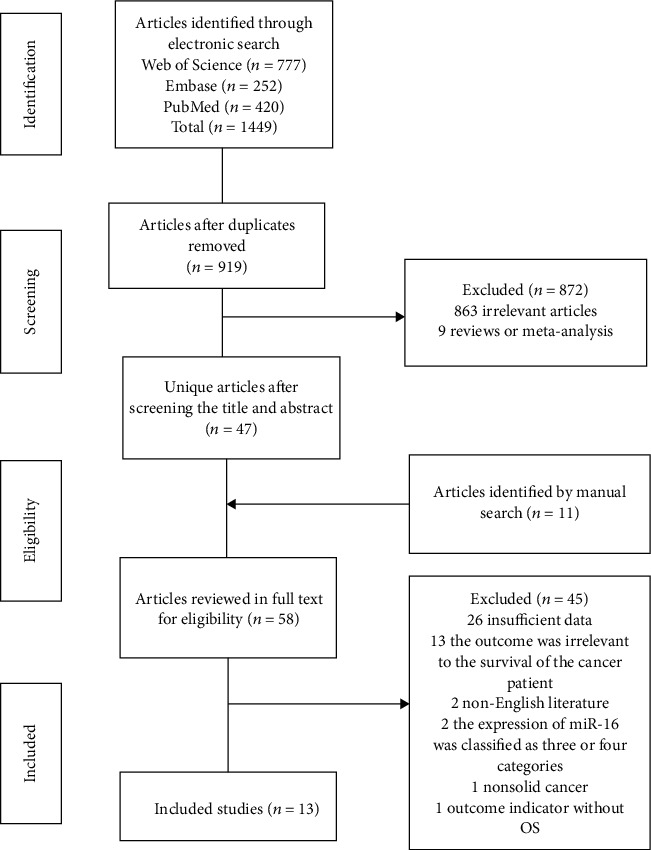
Flowchart of the selected studies (*n* = 13).

After duplicate checking, a total of 919 articles were identified by a literature search. There were 47 articles identified after screening the title and abstract. Review references were searched manually, and we added 11 articles for full-text reading. According to inclusion and exclusion criteria, 45 articles were excluded and a total of 13 studies were included [14–19, 27–33]. There were 2303 patients involved in this meta-analysis. The flowchart of literature screening is shown in Figure 1. The highest quality score was 19 points, and the lowest score was 8. Bounded by the median, there were 7 studies with a quality score of more than 15 points. Characteristics of the included studies are presented in Table 1.

### 3.2. Association between miR-16 Expression Level and Prognosis of Solid Cancer Patients by Meta-Analysis

**Figure 2:**
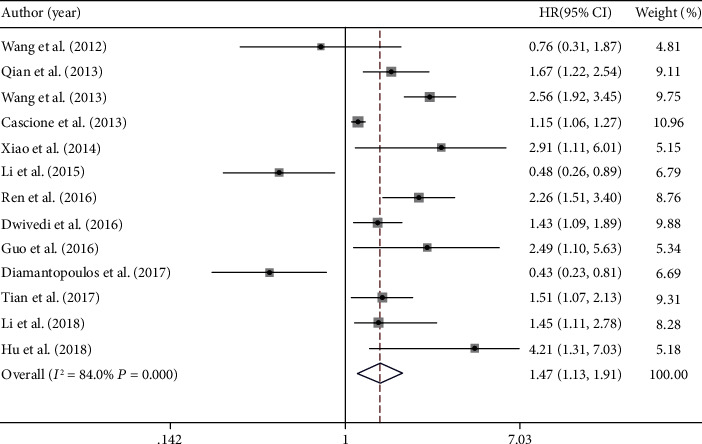
Forest plot for the association between miR-16 expression level and prognosis of solid cancer patients.

There was large interliterature heterogeneity among the studies (*I*^2^ = 84.0%, *P* ≤ 0.001). In the random effects model, the pooled HR was 1.47 (95% CI: 1.13-1.91, *P* = 0.004), indicating that the survival of cancer patients with low miR-16 expression level had a worse prognosis than that of those with high miR-16 expression level, and the results were statistically significant (Figure 2).

According to subgroup analyses stratified by publication year, there was a significant association between miR-16 expression level and prognosis of cancer patients in the studies published in 2014 and before (*I*^2^ = 88.3%, HR = 1.63, 95% CI: 1.04-2.57, *P* = 0.034). In the stratified analyses of cancer sites, low miR-16 expression level was associated with poor prognosis in the reproductive system cancers (*I*^2^ = 33.3%, HR = 1.24, 95% CI: 1.06-1.45, *P* = 0.008) and other system cancers (*I*^2^ = 63.5%, HR = 2.07, 95% CI: 1.38-3.10, *P* ≤ 0.001). However, the association between miR-16 expression level and prognosis of digestive system cancers was not statistically significant. In terms of regions, the three geographic locations presented inconsistent results. In Asian studies, lower miR-16 expression level was associated with poor prognosis (*I*^2^ = 71.7%, HR = 1.62, 95% CI: 1.15-2.29, *P* = 0.006) and marginally associated with worse prognosis among American studies (*I*^2^ = 92.8%, HR = 1.59, 95% CI: 1.00-2.53, *P* = 0.049). However, it was associated with favorable prognosis in European regions (HR = 0.43, 95% CI: 0.23-0.81, *P* = 0.009). The results were significant when the sample size was 100 to 199 (*I*^2^ = 82.8%, HR = 1.63, 95% CI: 1.15-2.30, *P* = 0.006) and ≥200 (*I*^2^ = 78.2%, HR = 1.77, 95% CI: 1.17-2.67, *P* = 0.007), while the result was opposite when the sample size was <100. After the quality score was divided into 15 boundaries, a significant association between miR-16 expression level and cancer prognosis was shown in the high-quality studies (*I*^2^ = 86.9%, HR = 1.50, 95% CI: 1.03-2.19, *P* = 0.036). When the studies were stratified by the statistical methods, we found that the result of the Cox model was statistically significant (*I*^2^ = 85.9%, HR = 1.64, 95% CI: 1.17-2.30, *P* = 0.004); nevertheless, the significant association was not found by the log-rank model. In the subgroup analysis of the biosample, low expression of miR-16 was associated with unfavorable prognosis in tissue samples (*I*^2^ = 80.0%, HR = 1.46, 95% CI: 1.11-1.93, *P* = 0.008) (Table 2).

**Figure 3:**
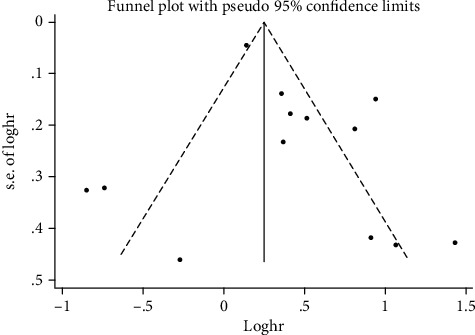
Begg's funnel plot of the included studies for the association between miR-16 expression level and prognosis of solid cancer patients.

After all the included studies were successively removed, the results were statistically significant with pooled HR values ranging from 1.39 (95% CI: 1.08-1.78) to 1.61 (95% CI: 1.24-2.08). By drawing funnel plots (Figure 3), it could be intuitively observed that the scatter distribution on both sides was relatively symmetric. The *P* values of Begg's test and Egger's test were 0.760 and 0.269, proving that there was no significant publication bias.

### 3.3. Survival Analysis of Solid Cancers through the miRpower Database

The survival analyses by the miRpower database included various types of solid cancer with 7642 patients to verify the results of this meta-analysis. After setting the median as the cutoff value distinguishing high and low mir-16 expression levels, there were statistically significant associations of mir-16 expression with pancreatic ductal adenocarcinoma (HR = 1.67, 95% CI: 1.10-2.56, *P* = 0.015) and thymoma (HR = 7.69, 95% CI: 0.97-50.0, *P* = 0.022) survival. However, the inverse association was found in liver hepatocellular carcinoma (HR = 0.68, 95% CI: 0.48-0.96, *P* = 0.029) and sarcoma (HR = 0.65, 95% CI: 0.43-0.96, *P* = 0.031). Kaplan-Meier survival curves for solid cancer mentioned above are shown in Supplementary Figure 1.

After pooling the effect size of the association between the mir-16 expression level and specific cancer site, we found that low expression of miR-16 was associated with poor prognosis of solid cancers (HR = 1.10, 95% CI: 1.00-1.19, *P* = 0.033). The subgroup analysis stratified by cancer location showed that the association in the reproductive system was significant (HR = 1.21, 95% CI: 1.03-1.42, *P* = 0.017) (Table 3).

## 4. Discussion

Cancer incidence and mortality are rapidly growing worldwide, with an estimation of 18.1 million new cancer cases and 9.6 million cancer deaths that occurred in 2018 [34]. Tumorigenesis is a multistep process and a multifactorial pathology characterized by environmental risk factors and genetic alterations, which poses a challenge to the prevention and control. In recent years, it has become a hot spot to search for clinical, therapeutic, and prognostic markers of cancer at the molecular level. The miRNA is providing research direction for scholars, due to the characteristics of easy separation and stability, and it also plays an important role in the regulation of a large number of biological processes and diseases [35, 36].

A study has shown that the increased expression of miR-17 was associated with unfavorable cancer prognosis [37]. Meanwhile, several meta-analysis studies have investigated the association between certain miRNA and prognosis of lung cancer, prostate cancer, head and neck cancer, which identified some miRNAs with a prognostic value, such as miR-21, miR-155, and miR-18a [38–40]. However, the inconsistent conclusions about the association between the expression of miR-16 and prognosis of solid cancer patients have not been reviewed. As far as we know, this is the first meta-analysis to show the exact association between miR-16 expression and prognosis of solid cancer patients.

Overall, this meta-analysis suggested that low expression of miR-16 contributed to poor prognosis of solid cancer patients with high heterogeneity. The subgroup analyses showed that the cancer type might contribute to the heterogeneity partially because heterogeneity was reduced in reproductive cancers, which showed that high expression of miR-16 was more favorable for cancer prognosis. This suggested that the organs where cancer occurred might be the source of heterogeneity. Unfortunately, for other cancers, such as respiratory and nervous systems, the number of studies was small and the heterogeneity cannot be tested. The results of bioinformatic analysis showed that miR-16 expression level was significantly associated with the prognosis of pancreatic ductal adenocarcinoma, thymoma, liver hepatocellular carcinoma, and sarcoma. After pooling the results from the same system of solid cancers, we found that miR-16 expression level was associated with the prognosis of the reproductive cancers, which was consistent with our meta-analysis. Thus, bioinformatic analysis further validated the reliability of this meta-analysis.

The relationship between miR-16 expression level and prognosis of solid cancer patients in different regions was completely discrepant, which might be related to the expression difference caused by different ethnic groups. The complexity of patient characteristics could explain the difference. Furthermore, there was only one European study [18], and the number of American studies [15, 27, 32] was relatively small; therefore, more relevant studies should be supplemented for obtaining and confirming stable results. We also found that the association between miR-16 expression level and prognosis of solid cancer patients showed higher HRs among studies using the Cox model that was adjusted for the confounding factor than those using the log-rank test. Thus, the log-rank test without any adjustment for potential confounding factors decreased the HRs.

Previous evidence has revealed that the expression level of miRNA-16 is affected by several genetic factors. Calin et al. [41] showed that the chromosome 13q14 deletion was related to a downregulation of miR-16 and the pathogenesis of chronic lymphocytic leukemia (CLL). Some researchers [42, 43] found that histone deacetylases were overexpressed in CLL leading to the aberrant epigenetic silencing of miR-16 expression. miR-16 modulates the cell cycle, inhibits cell proliferation, promotes cell apoptosis, and suppresses tumorigenicity both in vitro and in vivo [44]. There are several hypotheses that could explain the mechanism of miR-16 expression in cancer prognosis. miR-16 inhibits FEAT that is faintly expressed in normal tissues and aberrantly overexpressed in tumors and consequently promoted the apoptosis of cancer cells [45]. You et al. [46] found that miR-16 recognizes the 3′-UTR of KRAS transcription directly and regulates KRAS expression inhibiting tumorigenesis negatively. miR-16 was likely to suppress cancer growth by regulating the expression of genes such as CDK1 and CDK2, which are associated with cell cycle control and cellular proliferation [47]. This effect of inhibiting tumor proliferation and metastasis was also shown in cancer targeting transcription factor Sal-like protein 4 (SALL4) [48]. At the same time, another study showed that miR-16 appeared to be a major regulatory factor in suppressing Wip1 protein expression, which was a critical inhibitor in the ATM/ATR-p53 DNA damage signaling pathway [49]. It was reported that miR-16 negatively regulated Bcl2 in chronic lymphocytic leukemia and prostate and hepatocellular carcinoma cancer cells [50–52]. In addition, miR-16 represses colorectal cancer cell growth in vitro by regulating the p53/survivin signaling pathway [53]. These pieces of evidence were consistent with our results that high expression of miR-16 is beneficial to patients' survival.

There were several limitations of our study. Firstly, the cutoff value for distinguishing high and low expression of miR-16 was diversiform in the included studies. Secondly, the storage and treatment of samples taken from tissues and plasma or serum were different, such as fresh samples, frozen in nitrogen tanks and made into formalin-fixed paraffin-embedded (FFPE) samples, which affected the stability of the results. Thirdly, the included studies were conducted among participants from three countries; our findings may be limited when extrapolated to other study populations with different ethnicities. Finally, only published literatures were included in this analysis, and several unpublished research results that met the inclusion criteria were lost. Meanwhile, the included studies were limited to English, and some related studies in other languages that might meet the inclusion criteria might be missed.

## 5. Conclusion

There were enough high-quality studies in this study, which could indicate that miR-16 had a potential value to become a prognostic marker in solid cancer patients. Subgroup analysis showed that low miR-16 expression level was associated with poor prognosis in the reproductive system cancers, while not in digestive system cancers, which was further validated by bioinformatic analysis.

## Figures and Tables

**Table 1:** Characteristics of the studies included in the meta-analysis.

Author	Year	Country	No. of patients	No. of males	Age at baseline (mean/median/range) (years)	Biosample	Position	Stage	Follow-up (mean/median/range) (month)	Score	Statistical method	Cutoff
Wang et al. [31]	2012	China	85	49	/57/23-84	Tissue	Colorectum	I-IV	/52/2-59	13	Cox model	Upper tertile
Qian et al. [29]	2013	China	143	92	NA	Tissue	Colorectum	I-IV	/57.4/2.2-108.4	14	Cox model	Best performing (0.52)^∗^
Wang et al. [32]	2013	USA	391	203	62.05//	Serum	Lung	III-IV	/10.3/1.0–85.7	16	Cox model	Median (0.71)
Cascione et al. [27]	2013	USA	173	0	/43/20-50	Tissue	Breast	I-IV	/79/9-194	16	Cox model	Comparison with corresponding adjacent normal tissue
Xiao et al. [16]	2014	China	126	76	/66/22-82	Tissue	Colorectum	I-IV	/74.16/12-120	15	Cox model	Median (1.93)
Li et al. [19]	2015	China	38	30	NA	Plasma	Esophagus	I-IV	/22/4-95	14	Log-rank	Median
Ren et al. [14]	2016	China	180	130	NA	Tissue	Stomach	I-IV	/85.2/79.2-97.2	13	Cox model	2-fold change
Dwivedi et al. [15]	2016	USA	216	0	NA	Tissue	Ovary	NA	NA	8	Log-rank	Best performing (0.41)
Guo et al. [28]	2016	China	120	53	51.59//	Serum	Skin	I-IV	39.32//	15	Cox model	Median (0.399)
Diamantopoulos et al. [18]	2017	Greece	182	95	67.5//	Tissue	Colorectum	I-IV	//≥132	15	Cox model	Best performing (5.05)
Tian et al. [30]	2017	China	132	84	/45/12-78	Tissue	Brain	NA	/20/5-50	16	Cox model	Best performing
Li et al. [33]	2018	China	386	0	//28-79	Serum	Breast	I-IV	/31/19-40	19	Cox model	Best performing (0.3)
Hu et al. [17]	2018	China	131	82	NA	Tissue	Oral	I-IV	NA	12	Cox model	Median

^∗^The optimal cutoff value was determined by plotting the receiver operating characteristic (ROC) curve.

**Table 2:** Pooled and subgroup analyses stratified by potential modifying factors on the association between miR-16 and overall survival (OS) of solid cancer patients.

Subgroup	No. of studies	HR (95% CI)	*P* value	*I* ^2^ (%)	*P* for heterogeneity	*P* in metaregression
Overall	13	1.47 (1.13-1.91)	0.004	84.0%	≤0.001	
Publication year						0.672
>2014	8	1.38 (0.92-2.06)	0.117	81.9%	≤0.001	
≤2014	5	1.63 (1.04-2.57)	0.034	88.3%	≤0.001	
Cancer site						0.360
Digestive system	7	1.32 (0.72-2.39)	0.368	85.7%	≤0.001	
Reproductive system	3	1.24 (1.06-1.45)	0.008	33.3%	0.224	
Other	3	2.07 (1.38-3.10)	≤0.001	63.5%	0.064	
Region						0.280
Asia	9	1.62 (1.15-2.29)	0.006	71.7%	≤0.001	
Europe	1	0.43 (0.23-0.81)	0.009	—	—	
America	3	1.59 (1.00-2.53)	0.049	92.8%	≤0.001	
Sample size						0.138
<100	2	0.55 (0.33-0.93)	0.025	0.0%	0.403	
100-199	8	1.63 (1.15-2.30)	0.006	82.8%	≤0.001	
≥200	3	1.77 (1.17-2.67)	0.007	78.2%	0.010	
Quality score						0.897
<15	6	1.44 (0.92-2.25)	0.109	80.2%	≤0.001	
≥15	7	1.50 (1.03-2.19)	0.036	86.9%	≤0.001	
Statistical method						0.312
Cox mode	10	1.64 (1.17-2.30)	0.004	85.9%	≤0.001	
Log-rank	3	1.09 (0.64-1.85)	0.747	81.9%	0.004	
Biosample						0.986
Tissue	9	1.46 (1.11-1.93)	0.008	80.0%	≤0.001	
Serum or plasma	4	1.46 (0.71-2.99)	0.300	87.7%	≤0.001	

HR: hazard ratio; CI: confidence interval.

**Table 3:** HRs and 95% CIs of solid cancer patients with low miR-16 expression level in a Kaplan-Meier plotter database.

Cancer types	Sample size	HR (95% CI)	*P* value
Total	7642	1.10 (1.00-1.19)	0.033
Digestive system			
EAC	89	0.70 (0.39-1.27)	0.237
ESCC	95	0.56 (0.26-1.22)	0.143
LIHC	371	0.68 (0.48-0.96)	0.029
PAAD	178	1.67 (1.10-2.56)	0.015
READ	160	1.00 (0.46-2.17)	0.991
STAD	431	1.15 (0.85-1.56)	0.353
Subtotal	1324	0.93 (0.66-1.30)	0.682
Reproductive system			
BRCA	1076	1.22 (0.88-1.69)	0.230
CSCC	307	1.52 (0.94-2.44)	0.081
OC	485	1.09 (0.86-1.35)	0.495
TGCT	134	0.97 (0.14-7.14)	0.978
UCEC	537	1.49 (0.97-2.27)	0.065
Subtotal	2539	1.21 (1.03-1.42)	0.017
Urinary system			
BLCA	408	1.23 (0.92-1.67)	0.161
KIRC	516	0.93 (0.68-1.25)	0.613
KIRP	290	0.95 (0.53-1.72)	0.880
Subtotal	1214	1.06 (0.86-1.29)	0.583
Respiratory system			
LUSC	472	1.12 (0.85-1.49)	0.434
LUAD	504	1.15 (0.86-1.54)	0.347
Subtotal	976	1.13 (0.93-1.39)	0.222
Other system			
HNSC	522	1.13 (0.93-1.39)	0.053
THCA	506	1.47 (0.54-4.00)	0.450
PCPG	179	0.22 (0.03-1.96)	0.138
SARC	259	0.65 (0.43-0.96)	0.031
THYM	123	7.69 (0.97-50.0)	0.022
Subtotal	1589	1.08 (0.95-1.23)	0.810

BLCA: bladder carcinoma; BRCA: breast cancer; CSCC: cervical squamous cell carcinoma; EAC: esophageal adenocarcinoma; ESCC: esophageal squamous cell carcinoma; HNSC: head-neck squamous cell carcinoma; KIRC: kidney renal clear cell carcinoma; KIRP: kidney renal papillary cell carcinoma; LIHC: liver hepatocellular carcinoma; LUAD: lung adenocarcinoma; LUSC: lung squamous cell carcinoma; OC: ovarian cancer; PAAD: pancreatic ductal adenocarcinoma; PCPG: pheochromocytoma and paraganglioma; READ: rectum adenocarcinoma; SARC: sarcoma; STAD: stomach adenocarcinoma; TGCT: testicular germ cell tumor; THYM: thymoma; THCA: thyroid carcinoma; UCEC: uterine corpus endometrial carcinoma.

## Data Availability

All data generated or analyzed during this study are included in this article.

## Supplementary Materials

Supplementary Materials Supplementary Table 1: The PRISMA checklist for this meta-analysis describing the page in which every item is located. Supplementary Figure 1: Kaplan-Meier survival curves for solid cancer patients using the median as the cutoff value, including pancreatic ductal adenocarcinoma (a) and thymoma (b), liver hepatocellular carcinoma (c), and sarcoma (d).
